# [Be(NH_3_)_16_]^[2]^^+^ Microsolvation: Structure, Energetics, and Temperature Effects

**DOI:** 10.1002/cphc.202500654

**Published:** 2025-11-03

**Authors:** Awatef Hattab, Alhadji Malloum, Jeanet Conradie, Zoubeida Dhaouadi, Nino Russo

**Affiliations:** ^1^ Laboratoire de Spectroscopie Atomique Moléculaire et Applications Faculté des Sciences de Tunis Université de Tunis El Manar Campus Universitaire 1060 Tunis Tunisie; ^2^ Faculté des Sciences de Bizerte Université de Carthage 7023 Zarzouna Bizerte Tunisie; ^3^ Department of Chemistry University of the Free State Bloemfontein South Africa; ^4^ Department of Physics Faculty of Science The University of Maroua POBOX46 Maroua Cameroon; ^5^ Dipartimento di Chimica e Tecnologie, Chimiche Universitá della Calabria ViaP. Bucci 87036 Rende(CS) Italy

**Keywords:** binding energies, cluster, Gibbs energies, MP2, temperature dependence

## Abstract

Gas‐phase structural, energetic, and thermal properties of the [Be(NH_3_)_16_]^2^ cluster are investigated by using the MP2/6‐311++G** level of theory. The relative stability of isomers is explained evidencing the long‐range electrostatic interactions and the spatial arrangement of NH_3_ ligands around Be^2^^+^ cation. The computed isomers binding strength and energies values are compared with that with beryllium cation coordinated from *n* =  1–6 ligands. A fitting approach yields an asymptotic binding energy of –32.2 kcal mol^−1^. Clustering energies suggest a compact and strongly bound first solvation shell, with weaker, secondary interactions beyond four to five ligands. The cluster thermal behavior is probed through temperature‐dependent solvation enthalpies (Δ*H*) and free energies (Δ*G*) in gas phase. Results show that Δ*G* slightly decreases with temperature, while Δ*H* increases, emphasizing the role of entropy in thermal stabilization. Finally, Quantum Theory of Atoms in Molecules analysis reveals the coexistence of Be^2^^+^—N coordination bonds and a network of N—H…N hydrogen bonds. These cooperative noncovalent interactions significantly enhance both structural and energetic stability.

## Graphical abstract

Microsolvation properties of [Be(NH_3_)_16_]^2+^ cluster.

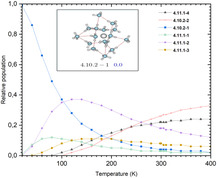

## 1 Introduction

The solvation behavior of charged alkaline earth metal ions, particularly the beryllium cation (Be^2^^+^), is of significant interest due to its important role in coordination chemistry, ion–solvent interactions, and the thermodynamic properties of ionic clusters in chemical,^[^ ^1^, ^2^, ^3^ ^]^ biological,^[^ ^4^, ^5^, ^6^, ^7^, ^8^ ^]^ and industrial systems.^[^ ^9^, ^10^, ^11^, ^12^, ^13^ ^]^ In particular, the coordination of Be^2^^+^ in ammonia has been extensively investigated using both theoretical and experimental approaches.^[^ ^14^, ^15^, ^16^, ^17^, ^18^, ^19^, ^20^, ^21^, ^22^, ^23^, ^24^, ^25^, ^26^ ^]^ Car–Parrinello molecular dynamics simulations involving 67 ammonia molecules around a Be^2^^+^ ion revealed a tetrahedral first solvation shell with four NH_3_ ligands directly coordinated and with Be^2^^+^—N bond lengths ranging from 1.694 to 1.849 Å.^[^ ^20^ ^]^ These values are slightly longer than those coming from the NMR, X‐ray diffraction, and neutron scattering experimental studies by Kraus et al. Their measurements indicated near‐tetrahedral geometry with a Be^2^^+^—N distances of 1.725–1.733 Å (X‐ray)^[^ ^26^ ^]^ and 1.710–1.740 Å (neutron diffraction)^[^ ^25^ ^]^ and a N—Be^2^^+^—N bond angles between 108° and 110°.

In our previous theoretical investigation using the MP2/6‐311++G(d,p) level of theory,^[^ ^27^ ^]^ we examined the solvation of Be^2^^+^ in ammonia, methanol, and water. For the [Be(NH_3_)_*n*_]^2^^+^ clusters, we showed that the second solvation shell could accommodate up to 12 ammonia molecules and that the inclusion of explicit solvent molecules in this outer shell led improve the agreement with the experimental data, underlining the importance of long‐range electrostatic interactions.

A parallel study of aqueous solvation demonstrated that the second solvation shell of [Be(H_2_O)_*n*_]^2^^+^ can host up to eight water molecules. Notably, the gas‐phase structure of [Be(H_2_O)_**1**_ **_2_**]^2^^+^ revealed that its most stable isomer lacks a fully saturated second shell.^[^ ^28^ ^]^ This suggests that in water the solvent molecules are preferentially localized in the outer shell where steric repulsion is reduced and hydrogen bonding is more favorable.

Despite these advances, experimental thermodynamic data such as Gibbs free energies (Δ*G*) and enthalpies (Δ*H*) for [Be (NH_3_)] ^2^^+^ clusters remain unavailable. This is mainly due to the high toxicity of beryllium^[^ ^3^, ^27^, ^28^, ^29^, ^30^, ^31^ ^]^ and the difficulty of handling unstable Be^2^^+^ species under laboratory conditions.^[^ ^1^, ^27^, ^28^, ^30^, ^32^ ^]^ In this context, quantum chemical calculations can play an important role in predicting structural and energetic properties.

To date, no study has examined the temperature dependence of isomer distributions in [Be(NH_3_)_*n*_]^2^^+^ clusters or assessed the effect of thermal variation on their stability. As a result, there is a lack of thermodynamic data for these systems under varying temperature conditions.

To address this gap, we have studied the structural, energetic, and thermodynamic characteristics of the [Be(NH_3_)_16_]^2^^+^ cluster employing the MP2/6‐311++G(d,p) ab initio level of theory. In addition, we analyzed the temperature‐dependent isomer distributions across the 20–400 K range. Furthermore, the binding and clustering energies have been computed. Moreover, solvation free energies (Δ*G*) and enthalpies (Δ*H*) were evaluated as functions of temperature, offering new insights into the thermal behavior of beryllium–ammonia clusters. Finally, the nature of the Be^2^^+^–ligand interactions was characterized using the Quantum Theory of Atoms in Molecules (QTAIM), revealing both coordination and hydrogen bonding interactions that collectively stabilize the cluster.

## 2 Methodology

All geometry optimizations and frequency calculations were performed using the Gaussian 16 software,^[^ ^33^ ^]^ employing a “tight” convergence criterion and an ultrafine integration grid to enhance numerical accuracy.

Initial configurations of the [Be(NH_3_)_1__6_]^2^^+^ clusters were generated using the ABCluster global optimization algorithm, originally developed by Zhang and Dolg.^[^ ^34^, ^35^ ^]^ These structures were then fully optimized at MP2/6‐311++G** level of theory. To ensure precise convergence during geometry optimizations, the following thresholds (in atomic units) were applied: root mean square (RMS) force =  1×10^−^^5^, maximum force =  1×10^−^^5^, RMS displacement =  4×10^−^^5^, and maximum displacement =  6×10^−^^5^.

Harmonic vibrational frequency analyses, carried out at the same level of theory, confirmed that all optimized geometries correspond to true minima on the potential energy surface, as indicated by the absence of imaginary frequencies. These frequency calculations also allowed for the determination of thermodynamic parameters, such as the Gibbs free energies and enthalpies of the microsolvated Be^2^^+^ clusters.

Basis set superposition error has been neglected since in our previous works on similar systems it has been found to be negligible.^[^ ^27^, ^28^, ^36^ ^]^

Further methodological details regarding the structural sampling procedure can be found in the foundational works of Zhang and Dolg,^[^ ^34^, ^35^ ^]^ as well as in our recent studies on solvation and microsolvation phenomena.^[^ ^27^, ^28^, ^36^, ^37^, ^38^, ^39^, ^40^, ^41^, ^42^, ^43^ ^]^

To evaluate the thermodynamic stability and relative isomers populations at a given temperature, canonical ensemble probabilities were computed using the Boltzmann distribution:

(1) $$P_{n}^{\left(k\right)}\left(T\right)=\frac{exp^{‐\beta G_{n}^{\left(k\right)}\left(T\right)}}{\sum_{k}exp^{‐\beta G_{n}^{\left(k\right)}\left(T\right)}}$$

where *β* =  $1/k_{B}$ *T*, with $k_{B}$ being the Boltzmann constant, and $G_{n}^{\left(k\right)}\left(T\right)$the free energy of the *k*th isomer of cluster size n at temperature *T*, as obtained from the Tempo code.^43^

The analysis considers temperatures of 25 K and above since quantum effects become increasingly significant below this threshold, potentially invalidating the assumptions of the classical canonical ensemble.

The isomers are designated using the *i*.*j*.*k* notation, where *i*, *j*, and *k* represent the number of ligand molecules located in the first, second, and third solvation shells, respectively. The sum *i + j* *+ k* equal the total number of ligands, *n*. When multiple isomers exhibit the same *i*.*j*.*k* distribution, they are indexed sequentially using the label *ijk‐m*, where *m* distinguishes between distinct structures within the same configuration.

The most stable structures considered for *n* =  1–6 in this work were extracted from our recent investigation on the solvation of beryllium in ammonia, carried out at the same level of theory.^[^ ^27^ ^]^

## 3 Results and Discussion

### 3.1 Structural Analysis versus Temperature of [Be(NH_3_)_16_]^2^^+^ Clusters

**Figure 1:**
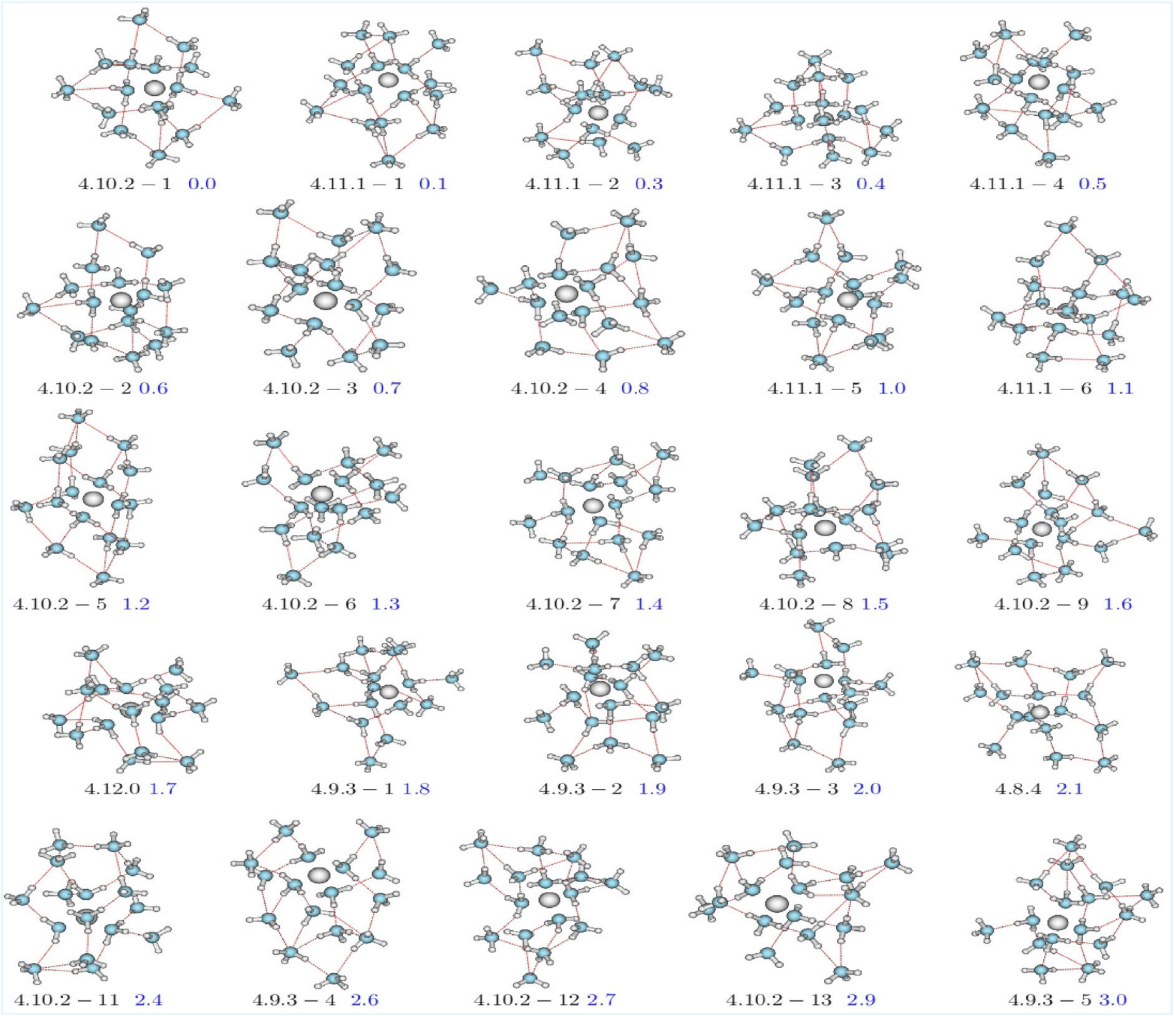
Gas‐phase structures of the most stable isomers of ${\left[\text{Be}{\left({\text{NH}}_{3}\right)}_{16}\right]}^{2+}$clusters at the MP2/6‐311++G(d,p) computational level. See Methodology section for the cluster definitions.

**Figure 2:**
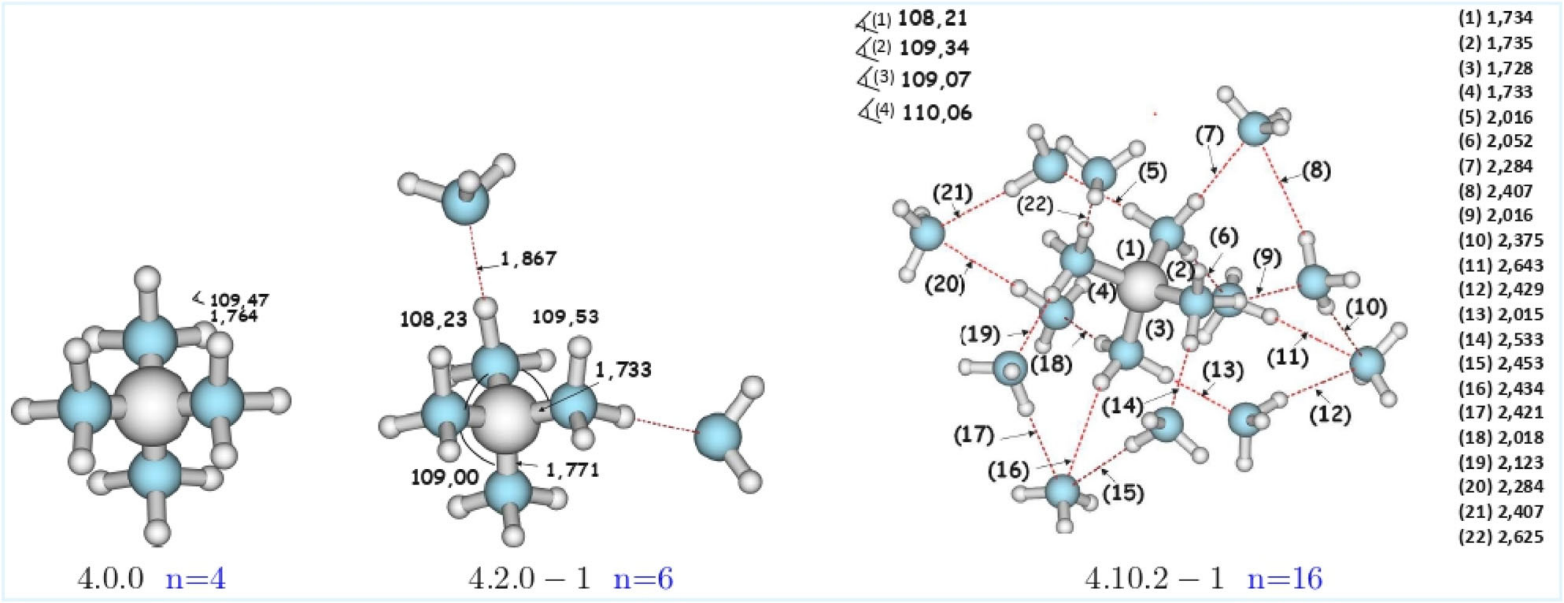
Geometries of ${\left[\text{Be}{\left({\text{NH}}_{3}\right)}_{n}\right]}^{2+}\text{system}$, (*n* =  4, 6, 16), optimized at MP2/6‐311++G(d,p).

Different isomers were identified for the [Be(NH_3_)_16_]^2^^+^ cluster (**Figure** **1**). The most stable, labeled 4.10.2‐1, features two ammonia molecules occupying the third solvation shell. This compact geometry is characterized by a Be^2^^+^—N bond length of 1.728–1.735 Å and an N—Be^2^^+^—N angle of 108.2°–110° (**Figure** **2**), values consistent with experimental data.^[^ ^20^, ^25^, ^26^ ^]^

This result is not unexpected and confirms findings from our previous study on the hydration of beryllium,^[^ ^28^ ^]^ in which the most stable structures no longer corresponded to those with a fully occupied second solvation shell, but rather to those incorporating one or more solvent molecules in the third shell. These results suggest the important role of long‐range interactions in stabilizing such complexes.

In addition to the global minimum, six low energy isomers (labeled 4.11.1‐1 through 4.11.1‐6) were identified, lying in an energy range of only 0.1–1.1 kcal mol^−1^ above the lowest‐energy isomer (Figure 1). In particular, the 4.11.1‐1 is almost isoenergetic with the global one and shows a Be^2^^+^—N bond length of 1.733 Å and a N—Be^2^^+^—N valence angle of 109.3°.

Thirteen additional isomers belonging to the 4.10.2‐m series (*m* =  1–13) were also characterized, with relative energies ranging from 0.6 to 2.9 kcal mol^−1^. These energetic variations are closely linked to the spatial arrangement of solvent molecules and the steric effects around the cation, both of which significantly influence cluster stability.

Isomer 4.12.0, featuring a fully filled second solvation shell (by 12 NH_3_ molecule) but lacking solvent molecules in the third shell, lies 1.7 kcal mol^−1^ above the global minimum. Its lower stability can be attributed to the absence of solvent molecules beyond the second solvation shell. In this structure, the Be^2^^+^—N bond length is 1.734 Å, slightly longer than in the global minimum (1.733 Å).

We note that the presence of ammonia molecules in the third coordination shell contributes to the shortening of Be^2^^+^—N bond lengths, in line with experimental findings,^[^ ^26^ ^]^ reinforcing the importance of long‐range electrostatic stabilization as previously reported.^[^ ^14^, ^27^, ^28^ ^]^

Additional high‐energy isomers (4.9.3‐1 to 4.9.3‐5), lying 1.8–3.0 kcal mol^−1^ above the global minimum, exhibit lower stability that can be attributed to the elongation of hydrogen bond (HB) distances in [Be(NH_3_)_16_]^2^^+^ cluster. In the 4.9.3‐1 isomer, the Be^2^^+^—N bond length is found to be 1.735 Å that is slightly longer than in 4.12.0. The 4.8.4 isomer, in which four NH_3_ molecules occupy the second solvation shell lies at 2.1 kcal mol^−1^ above the global minimum and exhibits a Be^2^^+^—N bond length of 1.735 Å.

In summary, our results show that both long‐range interactions (including hydrogen bonding) and the 3D spatial arrangement of solvent molecules are key factors governing the stability of beryllium–ammonia clusters. These effects ultimately lead to Be^2^^+^—N bond distances that are in excellent agreement with experimental data.^[^ ^20^, ^25^, ^26^ ^]^

**Figure 3:**
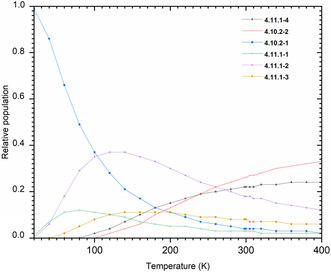
Temperature‐dependent of the relative populations of various isomers of ${\left[\text{Be}{\left({\text{NH}}_{3}\right)}_{n=16}\right]}^{2+}$in gas phase.

The temperature‐dependent isomer distribution of the [Be(NH_3_)_16_]^2^^+^ complex (**Figure** **3**) reveals that, among all structures located on its potential energy surface, only six isomers, namely, 4.10.2‐1, 4.10.2‐2, 4.11.1‐1, 4.11.1‐2, 4.11.1‐3, and 4.11.1‐4, contribute more than 2% to the total population over the studied temperature range. This is in line with our previous studies on temperature effects, which revealed that the isomers contributing significantly to the cluster population possess relative energies within ≈1.0 kcal mol^−1^.^[^ ^27^, ^28^, ^36^, ^37^, ^38^, ^39^, ^40^, ^41^, ^42^, ^43^ ^]^

At low temperatures, the 4.10.2‐1 isomer overwhelmingly dominates the distribution. Its population gradually decreases with increasing temperature, while the probabilities of isomers 4.10.2‐2 and 4.11.1‐*m* (*m* =  1, 2, 3, 4, and 8) exhibit the opposite trend, as shown in Figure 3. In particular, the 4.10.2‐2 isomer begins to appear around 100 K and its population steadily increases with temperature, reaching ≈33% at higher temperatures.

The isomers 4.11.1‐1 and 4.11.1‐2 reach maximum probabilities of nearly 12% and 38%, respectively, before decreasing at temperatures above ≈80 and ≈120 K.

Beginning around 40 K, the 4.11.1‐3 isomer starts to contribute to the population, although its probability remains below 10% throughout the range. Meanwhile, the 4.11.1‐4 isomer emerges at higher temperatures (above ≈80 K), and its population progressively increases, reaching ≈24% at elevated temperatures.

### 3.2 How Does the Second Solvation Shell Contribute to the Structural Stability and Hydrogen‐Bonding Network of [Be(NH_3_)_*n*_]^2^^+^ Clusters?

A comparative analysis of the [Be(NH_3_)_*n*_]^2^^+^ complexes for *n* =  4, 6, and 16 can help to understand as the progressive addition of NH_3_ molecules in the outer solvation shells influences both the stability and the geometric organization of the complex.

In the [Be(NH_3_)_4_]^2^^+^, the Be^2^^+^ cation adopts a nearly perfect tetrahedral geometry, characteristic of sp^3^ hybridization, with uniform Be^2^^+^—N bond lengths (≈1.764 Å) and coordination angles close to the ideal 109.5° value. This arrangement reflects a highly stable and symmetric environment, governed exclusively by direct coordination between the cation and the NH_3_ ligands.

The gradual addition of solvent molecules, as in the case of *n* =  6, introduces hydrogen‐bonding interactions between the directly coordinated ligands and the more distant NH_3_ molecules. These interactions have only a small effect on the geometry of the inner coordination sphere: the Be^2^^+^—N bond lengths remain in the range of 1.733–1.771 Å, and the Be^2^^+^ cation preserves its overall *sp* ^3^ coordination. This suggests that the surrounding NH_3_ molecules contribute to the cluster's stabilization through indirect effects, enhancing its energetic stability without significantly altering the geometry of the central structure.

In contrast, the larger *n* =  16 clusters exhibits a dense 3D hydrogen‐bonding network, with intermolecular distances ranging from 1.728 to 2.643 Å, clearly indicating the presence of three distinct coordination sphere. The Be^2^^+^—N bond lengths in this structure closely match the experimentally reported values (1.725–1.733 Å).^[^ ^26^ ^]^ In addition, a non‐negligible variation of the coordination angles around Be^2^^+^ is observed (from 108.2° to 110°) which falls within the experimental range (108°–110°).^[^ ^25^ ^]^ These variations reflect a slight distortion from the ideal sp^3^ tetrahedral geometry, caused by a combination of directional interactions, steric constraints, and cooperative effects among coordination spheres. The hydrogen‐bonding network not only stabilizes the complex but also influences the local geometry in agreement with experimental observations.

The data underline the critical role of extended solvation in the structural stabilization of [Be(NH_3_)_*n*_]^2^^+^ complexes. While the first coordination sphere provides rigid and symmetric bonding, the additional ammonia molecules influence the electronic environment and fine‐tune the local *sp* ^3^ geometry of the cation through noncovalent interactions, thereby enhancing the overall stability of the complex. These results demonstrate that, for a realistic representation of the structural and thermodynamic properties of solvated systems, it is essential to account for solvation beyond the first coordination sphere. In particular, the 4.10.2 configuration (*n* =  16) appears to be the most suitable model for studying Be^2^^+^ solvation in ammonia, as it closely reproduces experimentally observed geometric parameters.^[^ ^25^, ^26^ ^]^

### 3.3 Energetic Analysis of [Be(NH_3_)_1__6_]^2^^+^ Clusters

#### 3.3.1 Binding Energies of [Be(NH_3_)_1__6_]^2^^+^

The binding energy (Δ*E*) is defined as the energy required to form a given cluster from its individual components according to the following reaction:

(2) $${\mathrm{Be}}^{2+}+nNH_{3}\to {\left[Be{\left(NH_{3}\right)}_{n}\right]}^{2+}$$

Here, $E\left({\left[\text{Be}{\left({\text{NH}}_{3}\right)}_{n}\right]}^{2+}\right)$ and $E(NH_{3})$ represent the zero‐point corrected electronic energies of the cluster and the isolated ammonia molecule, respectively, while $E({\mathrm{Be}}^{2+})$refers to the electronic energy of the beryllium cation.

In order to evaluate the stability of the complexes on a per‐ligand basis, the binding energy per ammonia molecule (Δ*E*_*n*_ **)** was calculated using the most stable isomer identified for each cluster size *n* =  1–6 and *n* =  16 according to the following expression:

(3) $$\Delta E_{n}=\frac{\Delta E}{n}$$

**Figure 4:**
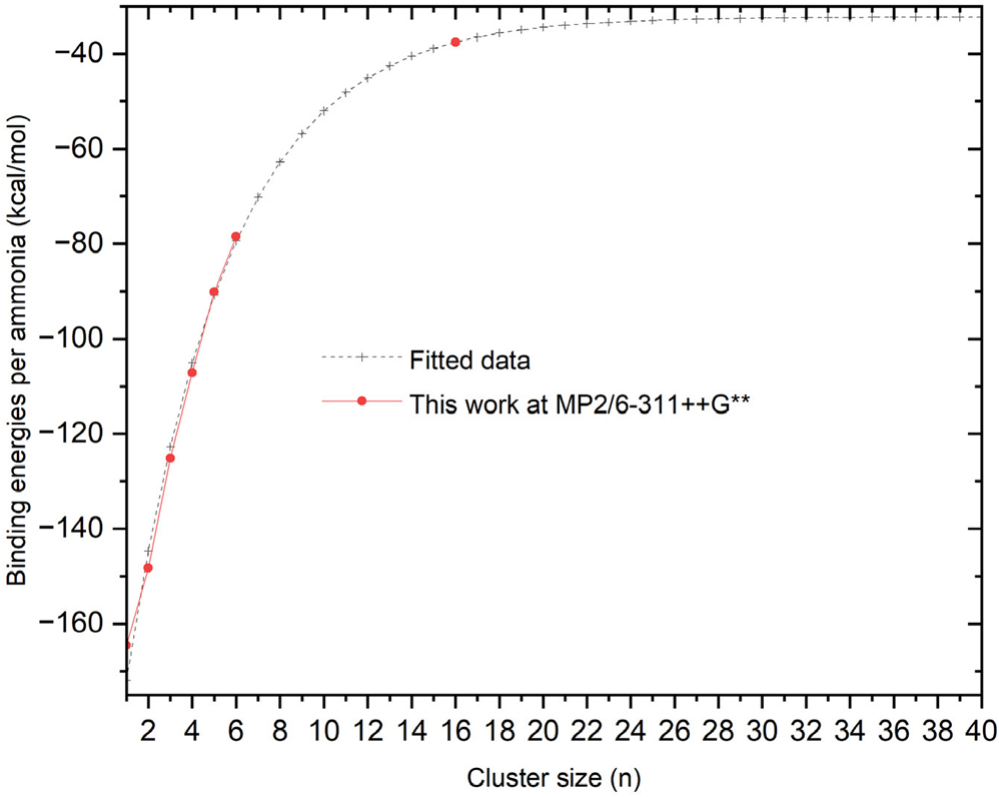
Fitted curve of the calculated binding energy of the ${\text{Be}}^{2+}$cation in ammonia molecules.

The resulting Δ*E*_*n*_ values, depicted in **Figure** **4**, suggest that the binding energy per ammonia molecule increases sharply for the first few ligands *n* =  1–4, reflecting strong interactions with the central beryllium ion and a progressive saturation of the first coordination shell around the Be^2^^+^ ion.

Beyond *n* =  4, the binding energy values increase slightly, indicating the onset of a second solvation shell. This outer shell is characterized by weaker interactions, primarily governed by hydrogen bonding networks, and long‐range interactions.

Therefore, it can be concluded that the first solvation shell of Be^2^^+^ in ammonia is essentially complete with four ligands as we reported in our previous works,^[^ ^27^ ^]^ while the subsequent ligands contribute mainly through secondary, less stabilizing interactions.

As the computed binding energies increase with cluster size, we performed a fit of our MP2/6‐311++G** data to analyze the convergence behavior. The variation of Δ*E*_*n*_ was modeled using the following exponential function:

(4) $$a_{1}+a_{2}e^{\frac{-x}{a_{3}}}$$

The optimized parameters obtained from the fit are: *a*_1_ =  –32.178423, *a*_2_ =  –173.54799, and *a*_3_ =  4.61323. The resulting fitted curve is shown in Figure 4. The mean absolute deviation between the fitted and computed binding energies is 0.1 kcal mol^−1^, and the correlation coefficient reaches *R* ^2^ =  0.9999, indicating an excellent agreement between the fitted function and our MP2 data.

Based on this analysis, we estimate that the binding energy Δ*E*_*n*_ for the [Be(NH_3_)_*n*_]^[2]^^+^ system converges to –32.2 kcal mol^−1^, which is effectively reached around *n* =  30.

#### 3.3.2 Clustering Energies of [Be(NH_3_)_*n*_]^2^^+^: Steric and Cooperative Effects

To gain a better understanding of the energetic behavior associated with the stepwise coordination of ammonia molecules around the Be^2^^+^ cation, we calculated the clustering energies (Δ*E_n_*‐_1_,*_n_*) of the [Be(NH_3_)*_n_*]^2^^+^ clusters at the MP2/6‐311++G** level of theory based on the following reaction:

(5) $${\left[\text{Be}{\left({\text{NH}}_{3}\right)}_{n-1}\right]}^{2+}+{\text{NH}}_{3}\to {\left[\text{Be}{\left({\text{NH}}_{3}\right)}_{n}\right]}^{2+}$$

**Figure 5:**
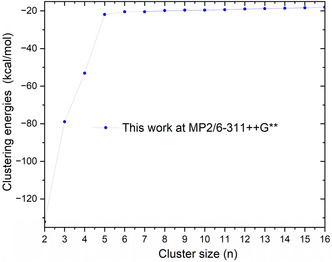
Clustering free energies of ${\left[\text{Be}{\left({\text{NH}}_{3}\right)}_{n=1-16}\right]}^{2+}$clusters as calculated from equation 4.

The results are presented in **Figure** **5**.

We emphasize that the fitted binding energy curve (Figure 4) served as the basis for estimating the energies of clusters with sizes *n* =  7–15, which were subsequently used in the calculation of clustering energies (Figure 5).

The curve reveals a steep increase of Δ*E* from *n* =  2 to *n *= 4, indicating strong coordination and significant stabilization as the initial ammonia molecules occupy the primary coordination sites around the Be^2^^+^ center. This energetic trend suggests the progressive formation of a first solvation shell up to *n* =  4, which corresponds to the optimal number of solvent molecules directly coordinating to the Be^2^^+^ center, as also supported by our previous study.^[^ ^27^ ^]^ At *n* =  5, a continued though increase in stabilization is observed (Figure 5), likely due the onset of interactions between the additional ligand sand those already present in the first shell, reflecting the strong coupling between inner‐ and outer‐shell ligands. Interestingly, at *n* =  6, a nonmonotonic variation appears, likely resulting from the interplay between two opposing effects: increasing ligand–ligand repulsion and additional stabilization from further coordination. Beyond *n* =  6, the clustering energies increase slightly and then converge toward a plateau at ≈–20 kcal mol^−1^, indicating that further ammonia molecules no longer coordinate directly to the Be^2^^+^ center, but provide only marginal stabilization.

This energetic plateau is consistent with the formation of a second solvation shell, where stabilization arises primarily from weaker noncovalent interactions, such as hydrogen bonding or dipole–dipole forces rather than direct metal–ligand coordination. These results highlight the critical role of the spatial arrangement of solvent molecules around the cation in determining the preferred coordination number and the solvation structure of Be^2^^+^, which are largely controlled by steric constraints and cooperative effects.

#### 3.3.3 Solvation Energies and Solvation Enthalpies versus Temperature of ${\left[Be{\left(NH_{3}\right)}_{n=16}\right]}^{2+}$

**Figure 6:**
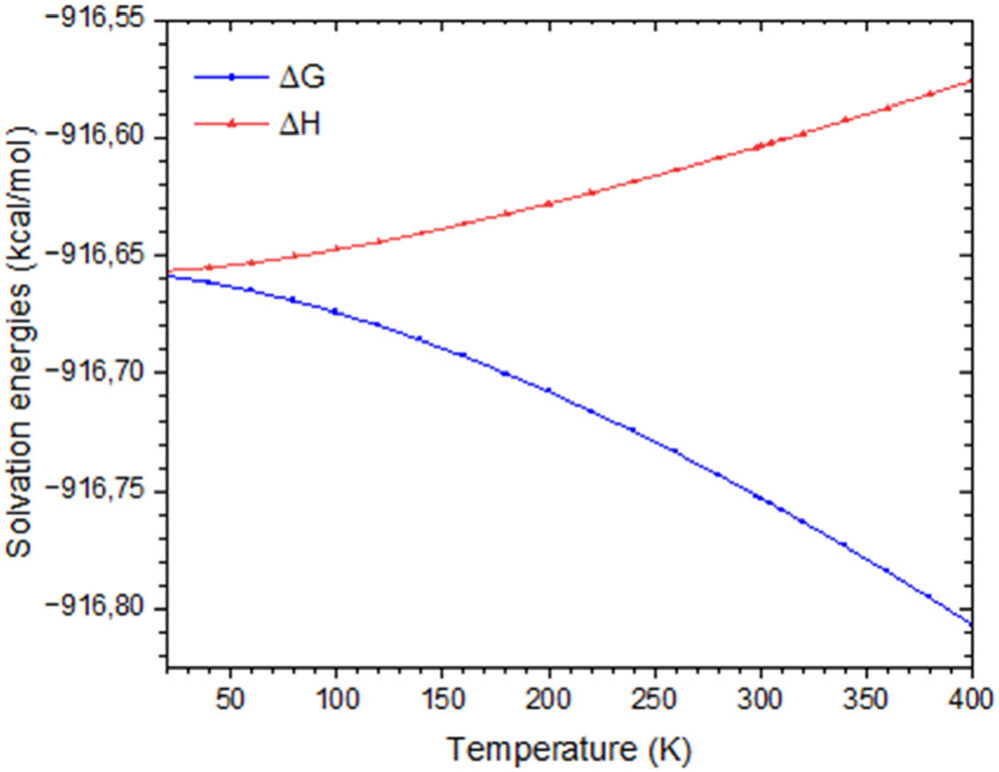
Solvation free energies versus solvation enthalpies in the gas phase for the 4.10.2‐1 isomer at different temperatures.

In this section, we examine the temperature dependence of the solvation enthalpy,$\Delta H_{\text{gas }}{\left({\text{Be}}^{2+}\right)}_{n}$, and solvation free energy, $\Delta G_{\text{gas }}{\left({\text{Be}}^{2+}\right)}_{n}$, of the ${\left[\text{Be}{\left({\text{NH}}_{3}\right)}_{n=16}\right]}^{2+}$ cluster in ammonia over the 20–400 K range (**Figure** **6**). To the best of our knowledge, these solvation energy values are reported here for the first time.

All data are referred to the most stable gas‐phase isomer for *n* =  16, identified as isomer 4.10.2‐1. As shown in Figure 6, the solvation free energy decreases slightly with increasing temperature, from –916.65 to –916.80 kcal mol^−^^1^, with an average slope of 0.4 cal mol^−^^1^ K^−^^1^. In contrast, the solvation enthalpy shows a slight increase, from –916.65 to –916.56 kcal mol^−^^1^, corresponding to an average variation of 0.2 cal mol^−^^1^ K^−^^1^.

This inverse temperature dependence of Δ*G* and Δ*H* suggests that, at low temperatures, solvation is primarily enthalpy‐driven due to strong ion–solvent interactions. At higher temperatures, entropy becomes the dominant contributor to stabilization, likely due to increased molecular disorder and the partial release of weakly bound ammonia ligands.

We conclude that the thermodynamic stability of solvated${\left[\text{Be}{\left({\text{NH}}_{3}\right)}_{n=16}\right]}^{2+}$ clusters at elevated temperatures is largely governed by entropic effects, while the enthalpic component remains nearly constant. This decoupling between enthalpy and entropic contributions has been reported in various solvation systems^[^ ^28^, ^39^, ^44^ ^]^ and likely represents a general feature of ion–solvent interactions.

#### 3.3.4 QTAIM Analysis of Noncovalent Bondings

In order to further elucidate the nature of the interactions between the beryllium cation and the coordinating ammonia molecules, a topological analysis of the electron density was performed using the QTAIM analysis,^[^ ^45^ ^]^ as implemented in the AIMAllprogram.^[^ ^46^ ^]^ The analysis was conducted on the most stable structure (4.10.2‐1) of the [Be^2^^+^(NH_3_)_16_] cluster.

Within the QTAIM approach, the electron density is examined through the identification of critical points, which are defined as positions in space where the gradient of the electron density vanishes. The nature of each critical point is determined by the eigenvalues of the Hessian matrix of the electron density and is classified as follows: (3, –3) atomic critical points, (3, –1) bond critical points (BCPs), (3, +1) ring critical points, and (3, +3) cage critical points.

This methodology not only confirms the presence and topology of chemical bonds but also allows for their quantitative characterization through the values of the electron density (*ρ*) and its Laplacian (∇^2^ *ρ*)^[^ ^47^, ^48^ ^]^ at the BCPs. These parameters provide key insights into the nature of bonding, enabling a clear distinction between covalent, dative, and noncovalent interactions such as hydrogen bonding.

**Figure 7:**
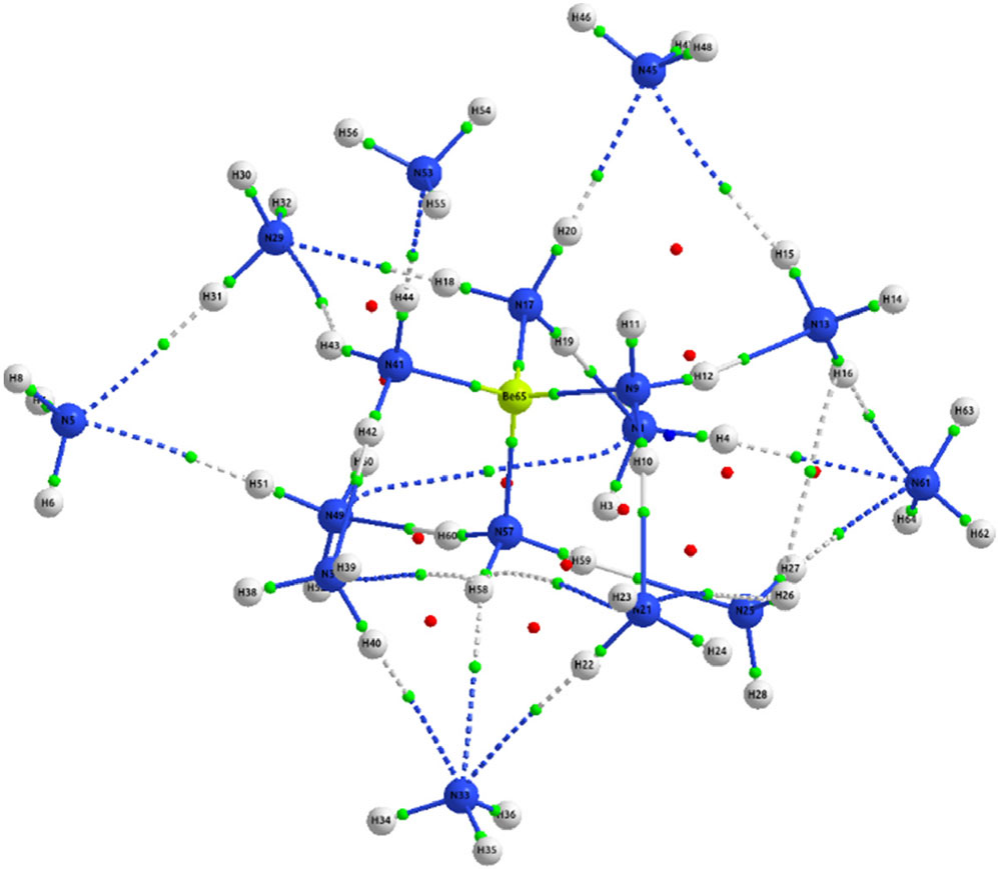
Critical points and bond paths of the most stable structure 4.10.2‐1 of [Be^2^^+^(NH_3_)_16_] complex.

According to the QTAIM analysis, reported in **Table** **1** and **Figure** **7**, the extreme values of the electron density (*ρ*) and its Laplacian (∇^2^ *ρ*) at BCPs focus exclusively on noncovalent interactions. From this table, it is evident that for NH···N HBs, the electron density at the BCPs ranges from 0.0020 to 0.0277 e Bohr^−^^3^, accompanied by positive Laplacian values between +0.0078 and +0.0784 e Bohr^−^^5^. These values are indicative of weak, closed‐shell interactions. In contrast, the Be···N dative bonds display considerably higher electron densities (*ρ* ≈ 0.0672–0.0690 e Bohr^−^^3^) and significantly larger positive Laplacian values (∇^2^ *ρ* ≈ +0.3885 to +0.3926 e Bohr^−^^5^), pointing to stronger and more polarized interactions with a partial covalent character. This contrast clearly illustrates the fundamental differences between hydrogen bonding and dative coordination in the [Be(NH_3_)_16_]^2^^+^ cluster. Further quantitative details on all BCPs are provided in the Supporting Information.

**Table 1:** Extreme values of the electron density (*ρ*) and its Laplacian (∇^2^ *ρ*) at BCPs corresponding to noncovalent interactions. The data refer to the most stable structure of the [Be^2^^+^(NH_3_)_1__6_] complex.

Bonding types	*ρ* [e Bohr^−^^3^]		∇^2^ *ρ*[e Bohr^−^^5^]	
	Min	Max	Min	Max
NH···N (H‐bond)	0.0020	0.0277	+0.0078	+0.0784
Be^2^^+^···N (dative bond)	0.0672	0.0690	+0.3885	+0.3926

## 4 Conclusion

In this work, we have investigated the structures, relative stabilities, and temperature‐dependent behavior of the [Be(NH_3_)_16_]^2^^+^ cluster in the gas phase at 0 K and over a temperature range from 25 to 400 K. All calculations were performed at the MP2/6‐311++G** level of theory. The results highlight that the key factors governing stability are long‐range interactions including hydrogen bonding and the 3D spatial arrangement of ammonia ligands, especially in geometries that minimize steric hindrance. Binding energies were computed for low‐lying isomers corresponding to *n* =  1–6 and *n* =  16. A reliable convergence trend of the binding energy per ligand was obtained through a fitting procedure, with high accuracy and without the need for additional computations. The results suggest that the influence of the Be^2^^+^ cation on the binding energy per ammonia molecule persists up to approximately *n* =  30, and we propose −32.2 kcal mol^−1^ as the limiting value at this level of solvation.

Clustering energies were also evaluated for the same range of cluster sizes. These data reveal that the primary solvation shell of Be^2^^+^ is essentially complete upon coordination with four ammonia ligands. Beyond this cluster size, additional ligands contribute mainly through secondary, weaker stabilizing interactions.

Furthermore, we examined the temperature dependence of the solvation enthalpy and free energy for the [Be(NH_3_)_16_]^2^^+^ cluster. Interestingly, Δ*H* and Δ*G* exhibit opposite temperature trends: while the solvation free energy decreases slightly with increasing temperature, the solvation enthalpy shows a slight increase. This behavior highlights the dominant role of entropic contributions in stabilizing the cluster at elevated temperatures.

To gain further insight into the bonding characteristics within [Be(NH_3_)_16_]^2^^+^, we performed a topological analysis of the electron density using the QTAIM. The QTAIM results reveal that the cluster is stabilized not only by direct coordination bonds between Be^2^^+^ and the nitrogen atoms of NH_3_ but also by an extensive network of intermolecular N—H…N HBs between the ammonia ligands. These cooperative noncovalent interactions play a critical role in both the structural organization and energetic stability of the solvation shell.

## Conflict of Interest

The authors declare no conflict of interest.

## Supporting information

Supplementary Material

## Data Availability

The data that support the findings of this study are available in the supplementary material of this article.
