# Supplementary material for: [Be(NH3)16][2] + Microsolvation: Structure, Energetics, and Temperature Effects
Source: Chemphyschem. 2025 Nov 3;26(24):e202500654. doi: 10.1002/cphc.202500654 (PMC12710208; doi:10.1002/cphc.202500654)
Supplement: Supplementary file 1 — Supplementary Material [file CPHC-26-e202500654-s001.pdf]

$[Be(NH_3)_{16}]^{[2]+}$ Microsolvation: Structure, Energetics, and Temperature Effects

Awatef Hattab[a,b], Alhadji Malloum[c,d], Jeanet Conradie[c], Zoubeida Dhaouadi[a,b], Nino Russo[e,*]

a) Laboratoire de Spectroscopie Atomique Moléculaire et Applications, Faculté des Sciences de Tunis, Université de Tunis El Manar, Campus Universitaire, 1060, Tunis, Tunisie.

b) Faculté des Sciences de Bizerte, Université de Carthage, 7023 Zarzouna Bizerte, Tunisie.

c) Department of Chemistry, University of the Free State, Bloemfontein, South Africa.

d) Department of Physics, Faculty of Science, The University of Maroua, PO BOX 46 Maroua, Cameroon.

e) Dipartimento di Chimica e Tecnologie Chimiche, Universitá della Calabria, Via P. Bucci, 87036 Rende (CS), Italy.

*Corresponding authors: nrusso@unical.it

SUPPORTING INFORMATION

Table S1 : Topological parameters at bond critical points (BCPs) for the N-H interactions in $[Be(NH_3)_{16}]^{2+}$ system. Listed values include electron density (ρ, e/ bohr^3) and its Laplacian ($\nabla^2\rho$, e/bohr^5), kinetic energy density (K, a.u.), ellipticity (ε), and the difference between bond path length and geometric bond length (BPL-GBL, Å).

BCP #	Name	Atoms	ρ	$\nabla^2\rho$	K	ε	BPL-GBL
1	BCP1	N1 - H19	0.025861	+0.074190	0.007723	-0.000550	0.000089
2	BCP2	N1 - H2	0.329444	-1.518846	0.034361	+0.446109	0.000061
3	BCP3	N1 - H3	0.329931	-1.508871	0.034853	+0.444719	0.000058
4	BCP4	N1 - H4	0.328885	-1.561281	0.033301	+0.454989	0.000064
5	BCP5	N5 - H51	0.011837	+0.034142	0.004679	-0.001051	0.017118
6	BCP6	N5 - H6	0.330531	-1.522295	0.034203	+0.447637	0.000057
7	BCP7	N5 - H7	0.329959	-1.535423	0.033819	+0.449632	0.000061
8	BCP8	N5 - H8	0.330331	-1.527012	0.034062	+0.448361	0.000058
9	BCP9	N5 - H31	0.015148	+0.045083	0.013709	-0.001347	0.012297
10	BCP10	N9 - H10	0.315564	-1.615896	0.020202	+0.460421	0.000037
11	BCP11	N9 - Be65	0.067379	+0.390206	0.004850	+0.006920	0.000139

12	BCP12	N9 - H11	0.328036	-1.524908	0.021332	+0.445508	0.000016
13	BCP13	H16 - N61	0.011129	+0.032021	0.061585	-0.000991	0.017805
14	BCP14	N9 - H12	0.314604	-1.610436	0.020273	+0.459014	0.000039
15	BCP15	H12 - N13	0.027769	+0.078441	0.012568	-0.000112	0.001737
16	BCP16	N13 - H14	0.329355	-1.522436	0.034503	+0.446752	0.000062
17	BCP17	N13 - H15	0.329765	-1.520765	0.034888	+0.447018	0.000063
18	BCP18	H4 - N61	0.011518	+0.033157	0.061780	-0.001016	0.017342
19	BCP19	N13 - H16	0.329094	-1.561900	0.033501	+0.455090	0.000063
20	BCP20	N17 - Be65	0.068969	+0.392626	0.005564	+0.008052	0.000107
21	BCP21	H15 - N45	0.005724	+0.016795	0.053253	-0.000490	0.055429
22	BCP22	N17 - H18	0.319491	-1.588158	0.019800	+0.455927	0.000027
23	BCP23	N17 - H19	0.316387	-1.598982	0.018904	+0.457245	0.000022
24	BCP24	N17 - H20	0.320193	-1.590896	0.018246	+0.456368	0.000019
25	BCP25	H10 - N21	0.027157	+0.077014	0.009942	-0.000262	0.000430
26	BCP26	N21 - H23	0.329693	-1.522525	0.034374	+0.447089	0.000060
27	BCP27	N21 - H22	0.329222	-1.555713	0.033655	+0.453892	0.000069
28	BCP28	N21 - H24	0.329673	-1.523231	0.034349	+0.447190	0.000059
29	BCP29	H27 - N61	0.010845	+0.030826	0.061065	-0.000930	0.016916
30	BCP30	H16 - H27	0.004085	+0.016782	8.512297	-0.000785	0.275977
31	BCP31	N21 - H26	0.002060	+0.007824	0.509648	-0.000422	0.358934
32	BCP32	N25 - H26	0.329788	-1.509342	0.034696	+0.444713	0.000059
33	BCP33	N25 - H27	0.329015	-1.560620	0.033065	+0.454830	0.000062
34	BCP34	N25 - H59	0.025122	+0.072519	0.009546	-0.000693	0.001159
35	BCP35	N25 - H28	0.329484	-1.515524	0.034323	+0.445495	0.000060
36	BCP36	H18 - N29	0.021720	+0.063395	0.019017	-0.001137	0.003919
37	BCP37	N29 - H30	0.329302	-1.503062	0.034061	+0.443249	0.000053
38	BCP38	N29 - H32	0.329163	-1.506297	0.034021	+0.443925	0.000052
39	BCP39	N29 - H31	0.326746	-1.575938	0.031794	+0.457182	0.000069
40	BCP40	N29 - H43	0.006937	+0.020387	0.084522	-0.000596	0.074197
41	BCP41	H22 - N33	0.011064	+0.032158	0.061370	-0.001016	0.021110
42	BCP42	N33 - H58	0.007543	+0.021096	0.064792	-0.000625	0.024633
43	BCP43	N57 - H58	0.326600	-1.547531	0.020985	+0.449334	0.000016
44	BCP44	N33 - H34	0.329660	-1.523798	0.033615	+0.447347	0.000056
45	BCP45	N33 - H35	0.329370	-1.530353	0.033359	+0.448384	0.000059

46	BCP46	N21 - H58	0.003297	+0.012939	4.035793	-0.000634	0.510194
47	BCP47	N57 - H59	0.316633	-1.606015	0.020460	+0.458533	0.000034
48	BCP48	N33 - H36	0.329998	-1.517497	0.033876	+0.446297	0.000057
49	BCP49	N33 - H40	0.012505	+0.036890	0.021897	-0.001161	0.017973
50	BCP50	N37 - H58	0.003908	+0.014268	1.449126	-0.000637	0.073365
51	BCP51	N37 - H38	0.329685	-1.525632	0.034105	+0.447681	0.000058
52	BCP52	N37 - H39	0.329801	-1.522274	0.034219	+0.447100	0.000057
53	BCP53	N37 - H42	0.027667	+0.077872	0.010922	-0.000130	0.000321
54	BCP54	N37 - H40	0.329110	-1.563466	0.033491	+0.455637	0.000069
55	BCP55	N57 - Be65	0.067322	+0.388517	0.004338	+0.007076	0.000088
56	BCP56	N41 - Be65	0.067256	+0.389016	0.006665	+0.006971	0.000013
57	BCP57	N41 - H42	0.315322	-1.614163	0.019852	+0.460062	0.000034
58	BCP58	H44 - N53	0.024516	+0.070907	0.010551	-0.000803	0.001446
59	BCP59	N41 - H43	0.327194	-1.542540	0.021714	+0.448726	0.000021
60	BCP60	N41 - H44	0.317166	-1.609993	0.019766	+0.459437	0.000030
61	BCP61	H20 - N45	0.020366	+0.060265	0.004143	-0.001303	0.002945
62	BCP62	N45 - H46	0.330030	-1.525706	0.033847	+0.447964	0.000059
63	BCP63	N45 - H47	0.329847	-1.529265	0.033761	+0.448448	0.000060
64	BCP64	N45 - H48	0.330203	-1.524213	0.034039	+0.447685	0.000060
65	BCP65	N1 - N49	0.002394	+0.009363	0.329550	-0.000512	0.211845
66	BCP66	N49 - H50	0.329899	-1.505278	0.034472	+0.443991	0.000055
67	BCP67	N49 - H51	0.327960	-1.571471	0.032307	+0.456407	0.000064
68	BCP68	N49 - H60	0.027192	+0.076916	0.010892	-0.000244	0.000360
69	BCP69	N49 - H52	0.329588	-1.518117	0.033962	+0.446104	0.000058
70	BCP70	N53 - H54	0.330518	-1.526872	0.033766	+0.448381	0.000057
71	BCP71	N53 - H55	0.330146	-1.533892	0.033467	+0.449462	0.000058
72	BCP72	N53 - H56	0.330061	-1.536393	0.033383	+0.449944	0.000058
73	BCP73	N57 - H60	0.315383	-1.610344	0.020667	+0.459213	0.000039
74	BCP74	N61 - H64	0.329626	-1.526679	0.032994	+0.447827	0.000055
75	BCP75	N61 - H62	0.329620	-1.526329	0.033026	+0.447752	0.000055
76	BCP76	N61 - H63	0.329603	-1.526260	0.033022	+0.447728	0.000055
